# Supplementary material for: Exploring the knowledge, attitudes, and practice towards child eye health: A qualitative analysis of parent experience focus groups
Source: PLoS One. 2023 Nov 3;18(11):e0293595. doi: 10.1371/journal.pone.0293595 (PMC10624311; doi:10.1371/journal.pone.0293595)
Supplement: S1 Table — (DOC) [file pone.0293595.s001.doc]

Supplementary Table 1. Details of Participants in Each Focus Group Discussion

| **Participant Address and Location of FGD** | **Sex** | | | | | | **Age Range of participants in each FGD** |  |
| --- | --- | --- | --- | --- | --- | --- | --- | --- |
|  |  |  |  |  |  |  |  |  |
|  | **Male** | | **Female** | | **Total** | |  |  |
|  | **n** | **%** | **n** | **%** | **n** | **%** |  |  |
| Aba Jale Kebele 11 | 11 | 15% | 2 | 3% | 13 | 18% | 27 to 68 years |  |
| Adebabay Iyesus 04 | 8 | 11% | 4 | 6% | 12 | 17% | 30 to 62 years |  |
| Shwa Ber Kebele 18 | 8 | 11% | 4 | 6% | 12 | 17% | 27 to 61 years |  |
| Azezo Kebele 20 | 8 | 11% | 1 | 1% | 9 | 13% | 32 to 61 years |  |
| Arbaya | 6 | 8% | 1 | 1% | 7 | 10% | 29 to 67 years |  |
| Worehala | 5 | 7% | 4 | 6% | 9 | 13% | 30 to 48 years |  |
| Kalay | 9 | 13% | 0 | 0% | 9 | 13% | 26 to 69 years |  |
| **Total** | **55** | **77%** | **16** | **23%** | **71** | **100%** | **26 to 69 years** |  |
