# Supplementary material for: Exploring the knowledge, attitudes, and practice towards child eye health: A qualitative analysis of parent experience focus groups
Source: PLoS One. 2023 Nov 3;18(11):e0293595. doi: 10.1371/journal.pone.0293595 (PMC10624311; doi:10.1371/journal.pone.0293595)
Supplement: S2 Table — (DOC) [file pone.0293595.s002.doc]

**Supplementary Table 2. Themes, subthemes and representative quotes**

|  | **THEMES** | **SUBTHEMES** | **REPRESENTATIVE QUOTES** |
| --- | --- | --- | --- |
| **KNOWLEDGE** | **1.0 Information on child eye health** | 1.1 Parents accessed child eye health (CEH) information from media, health professionals and patients. | "We get the information about eye health on television and radio." |
|  |  |  | "I am a religious leader. From what I observed, most of the time we get the information about the disease and eye care from a health center." |
|  |  |  | "Those who was treated and get cured from the illness tell us about it." |
|  |  |  | "Information on child eye health is obtained from health professionals working in under 5 children, and their treatment history." |
|  |  | 1.2 Content of CEH information was related to hygiene, optimal health visits, and avoiding bright lights and television. | "They received instruction on personal and environmental hygiene (particularly to washing hands frequently and keeping eyes clean)." |
|  |  |  | "Information on when to go to health facilities; avoiding bright light; and minimizing television time." |
|  |  |  | "Information on avoiding bright light; and minimizing television time." |
|  | **2.0 Knowledge about child eye problems** | 2.1 Causes of eye problems were many and varied | "When children see into the dark their eyes tend to deviate." |
|  |  |  | "We educate the society on vision decrement caused by trachoma." |
|  |  |  | "Reduction of vision could be due to trachoma, dirt, and a lot of other diseases." |
|  |  |  | "Deviation of the eyes in young children mostly arises from dirt, if they don’t was their faces two to three times per day." |
|  |  |  | "Visual decrement is usually caused by seeing water and glowing things." |
|  |  | 2.2 Signs of vision impairment in children included falling, difficulty reading from afar or seeing at night, and poor school performance. | "I personally have found many children who had squint, but I had no idea if it was treatable. And hence, I have not told them to seek treatment. But I know if they have decreased vision, it can be treated at hospital.” |
|  |  |  | "When our visual ability decreases, we have difficulties to read distant writings." |
|  |  |  | "Children fall when walking." |
|  |  |  | "Children in school can't properly copy." |
| **ATTITUDES** | **3.0 Barriers to child eye care** | 3.1 Financial constraints, distance and poor knowledge impede access to CEH services | "The major thing is due to a lack of knowledge - they don't think it can be treated." |
|  |  |  | "One is financial constraint, because of it many with curable disease failed to get treatment." |
|  |  |  | "Some who seek for traditional treatment because they cannot afford to go to a health center." |
|  |  |  | "Financial constraint, and most of them live far and there is no access for transportation." |
|  |  | 3.2 Long wait times and substandard quality of health institutions impede provision of CEH services | "Parents lose hope when they become appointed after visiting medical centers." |
|  |  |  | "Even at Gondar hospital there is very long appointment for services." |
|  |  |  | "The health centers is substandard - it will be like the pig story." |
|  |  |  | "Within the nearby hospital (Arbaya) [there is] no specialist eye doctor." |
|  | **4.0 Use of eye glasses** | 4.1 Eye glasses perceived to cause harm and stigmatize children | "Our tradition and thinking is that wearing glasses for children brings harm to them." |
|  |  |  | "When people see children wearing glasses they might say: '*Why is he wearing a glass? He's just a kid*'." |
|  |  |  | "They may also not play as much as the other kids cause of the fear that the glasses will break." |
|  |  |  | "Those children that wear glasses might be outcasted." |
|  |  |  | "If children wear their glasses and go to school they will be insulted and so they don’t wear [them]." |
|  |  | 4.2 Eye glasses perceived to improve vision | "Especially in students the impact it has, is being able to differentiate letters from a far and near." |
|  |  |  | "The treatment should continue as it is and glasses are mandatory for children." |
|  | **5.0 Impact of childhood blindness** | 5.1 Long term consequences include poor school performance and failure to progress in life | "Children may fall when walking if they have decreased vision." |
|  |  |  | "Economic difficulty is also another problem with blindness." |
|  |  |  | "Children can have be labeled as 'stupid'." |
| **PRACTICE** | **6.0 Prevention of blindness** | 6.1 Prevention of vision impairment includes having good personal hygiene, eating a healthy diet and avoiding sunlight | "To prevent reduction of vision the major thing is maintaining their hygiene." |
|  |  |  | "Eating a balanced diet (e.g. eating carrots was mentioned specifically) for preventing visual impairment." |
|  |  |  | "Avoiding reading in sunlight can prevent visual problems in children." |
|  | **7.0 Cultural practices & traditional medicine** | 7.1 Cultural terminology and practices | "'Alemochie’ means a word to describe when children develop fever, body rash, and red eyes." |
|  |  |  | "'Kindibat' is usually done by cutting the eyelid and it's treatment for red eye." |
|  |  |  | "Apply eye liners to newborn is one way to prevent eye diseases in children." |
|  |  | 7.2 Traditional medicine has a role in CEH | "Some people seek for traditional treatment because they cannot afford to go to health facilities." |
|  |  |  | "Some go towards traditional medicines." |
