# Supplementary material for: Exploring the knowledge, attitudes, and practice towards child eye health: A qualitative analysis of parent experience focus groups
Source: PLoS One. 2023 Nov 3;18(11):e0293595. doi: 10.1371/journal.pone.0293595 (PMC10624311; doi:10.1371/journal.pone.0293595)
Supplement: Attachment — Submitted filename: rev Exploring KAP -Child Eye Health_Manuscript.doc [file pone.0293595.s003.doc]

**Exploring the knowledge, attitudes, and practice towards child eye health: A qualitative analysis of parent experience focus groups**

Sadik Taju Sherief, ^1,5^ Samson Tesfaye, ^2^ Zelalem Eshetu,^3,^ ,Asim Ali^4^ , Helen Dimaras ^4,5^

^1^ Department of Ophthalmology, Addis Ababa University, Addis Ababa, Ethiopia;

^2^ Orbis International Ethiopia;

^3^ Biruh Vision Speciality Eye Center;

^4^ Department of Ophthalmology and Vision Sciences, The Hospital for Sick Children and University of Toronto, Toronto, Canada.

^5^ Child Health Evaluative Sciences Program and Centre for Global Child Health, Sickkids Research Institute, Toronto, Canada.

**Corresponding Author:**
Sadik T. Sherief, MD
Department of Ophthalmology
School of Medicine, College of Health Sciences
Addis Ababa University
P.O. Box 9086, Addis Ababa, Ethiopia
Tel: +251917801719
Email: goge4000@yahoo.com

**Short Title:** KAP towards CEH

ABSTRACT

**Background:** The majority of childhood blindness causes in low-income countries are treatable or avoidable. Parents or guardians are responsible for making decisions regarding a child's eye care. Understanding parents’ awareness and perception of eye problems is crucial in helping to know parents’ eye care-seeking behavior.

**Objective**: To report parents’ awareness and perception of eye diseases in their children.

**Methods:** Seven focus group discussions (FGDs) were carried out in North Western Ethiopia on knowledge, attitude and practice of parents towards child eye health. Their responses were tape-recorded and later transcribed. A thematic phenomenological approach was used for the analysis.

**Result:** A total of 71 parents participated in the FGDs. Participants were aware of common eye problems like trachoma, trauma, and glaucoma. However, they were unaware of the causes and etiologies of childhood blindness. Participants perceived that eye problems could be treated with hygiene and food, and often held misconceptions about the cause of strabismus and utilization of wearing spectacles.

**Conclusion:** The study revealed that parents are often unaware of the causes and etiologies of common childhood eye diseases, which has downstream effects on health-seeking behavior. Health promotion efforts, potentially through mass and social media, could be helpful to raise awareness, coupled with training of health professionals at primary and secondary health facility levels.

**Keywords:** child eye health, knowledge, attitudes, practice, pediatric ophthalmology

**INTRODUCTION**

Control of blindness in children is closely linked to child survival [1]. Without initiatives to stop preventable childhood blindness, it is anticipated that by 2020 the world would have lost more than US$110 billion [2]. In low-and-middle income countries, up to 60% of children die within a year of going blind – usually due to underlying conditions such as measles, meningitis, rubella, prematurity, genetic diseases and head injuries [3, 4]. In 2020, the estimated percentage of Gross Domestic Product (GDP) loss due to blindness in sub-Saharan Africa was 0.5% [5].

The prevalence of childhood blindness is closely linked to the socioeconomic development of the country and the mortality rate of those under five years of age [1, 6]. An estimated 19 million children are visually impaired globally, of which 1.26 million children are blind [7, 8]. Almost 3/4 of these children live in low-and-middle income countries (LMICs) [9], where there are limited data on prevalence and causes of childhood blindness. In sub-Saharan African countries the prevalence rate of child blindness is to be 1.5 per 1000 children, based on an extrapolation model using under five mortality rates [1, 9].

Early detection of childhood eye diseases at the household and community levels is essential in reducing the global burden of visual impairment and childhood blindness. However, poor parental awareness of common eye diseases and their prevention is one of the main challenges in the fight against childhood blindness [10-12]. In Africa, studies show that parental health seeking behavior is affected by cultural and social factors. For example, according to a widely held misconception, congenital blindness is thought to be incurable, and caused by ancestral curse [2].

The high prevalence of childhood visual impairment in sub-Saharan Africa has been attributed to lack of availability, accessibility, and affordability of eye care services [13]. The proper utilization of available eye care service is mandatory to reduce the burden of visual impairment worldwide [14]. Furthermore, studies have shown that common barriers to seeking eye examination in children include: the belief that eye examination is not necessary, limited finances, long distance between residence and the health facilities and the broader availability and lower cost of traditional medicine [15-18]. Literacy of parents is also a factor in accessing child eye health services; for example, in Malawi, parents who were illiterate were less likely to consent to their children having eye surgery [18].

The population of Ethiopia is estimated at 112,079,000 (2019). With the current 2.6% annual Rate of Natural Increase, the total population is projected to reach 144,944,000 by 2030 and double (205,411,000) by 2050 [20]. The prevalence of childhood blindness is 0.1% and accounts for over 6% of the total blindness burden [21]. Northwest Ethiopia in particular is more vulnerable to the burden of childhood blindness, as eye care facilities are limited in the area.

In order to improve the state of child eye health (CEH), policy makers would benefit from an understanding of the awareness, local context, perceptions, and beliefs of parents towards child eye care. The aim of this study was to assess knowledge, attitudes and practice among parents towards CEH in Northwest Ethiopia.

METHODS AND MATERIALS

**Study Design and Setting**

A qualitative study, using 7 focus group discussion (FGDs) was conducted in June 2022 in the North Gondar Zone of the Amhara region of Ethiopia, located 745 km northwest of Addis Ababa. The Zone has a total population of 3,999,113 with about a 1:1 male to female ratio [22]. Metema, Debark, and University of Gondar Hospital are three hospitals located within the Zone; the University of Gondar Hospital is the only tertiary eye care facility in the Zone. We used qualitative methods in order to obtain in depth insights of the knowledge, attitude and practice of parents towards CEH.

**Study Participants**

The participants included parents having children age less than 12 years of age. Participants were drawn from two Woredas (i.e., third level administrative districts) in the Central Zone of the Amhara region. To recruit a diverse set of study participants, study investigators sent an introductory study letter to each Woreda office asking for their support to help identify suitable candidates. Participants were purposively selected to ensure diverse representation of sociodemographic (i.e. sex, age, literacy, employment status) and employment type (e.g. health extension workers (HEW), farmers, teachers, religious or community leaders) where applicable.

**Data Collection**

Data were collected by two local health systems researchers, who received 2 days of training in qualitative data collection for the purpose of the study. Semi-structured topic guides were developed in English, translated into Amharic language and back-translated for consistency. The topic guides were piloted in a kebeles (i.e., smallest administrative districts in Ethiopia, akin to a neighborhood) that was not included in the study, and adaptations to questions were made. Demographic information (e.g. sex, age, kebele, religion, literacy, employment status, employment type) was collected for each participant prior to beginning the FGD. The FGD interview guide included questions on (i) knowledge (e.g. information about child eye care, knowledge about poor eyesight, barriers for accessing child eye care}, (ii) attitudes (e.g. towards spectacle wear) and (iii) practice (e.g. prevention of visual impairment, treatment and impact of visual impairment). Daily debriefing meetings with all data collectors were organized to examine major findings, review saturation, and refine research directions. All FGDs were tape recorded, transcribed and translated into English. The discussions lasted about 60 to 90 minutes and included 7-13 participants each.

**Data Analysis**

The transcripts were read to identify key themes and develop a coding framework. To do this, we first loaded the translated data into Open Code 4.03 (Umea University, Sweden), an open source computer software for analyzing qualitative data. We analyzed the data using a grounded theory approach, a systematic qualitative research methodology emphasizing generation of theory from data in the process of conducting research [23]. The coded transcripts were further analyzed and summarized into narratives for each theme and sub-theme.

**Ethical considerations**

The study was conducted after obtaining ethical clearance from the Addis Ababa University Institutional Review Board (Ref No. 039/22/Ophtha). Written informed consent was obtained from the participants at admission to participate in the research. And the study had no risk or negative consequence for those who participated. No personal identifiers were collected or used in the research report. Access to the collected information was limited to the principal investigator, and confidentiality was maintained throughout the project.

RESULTS

Study Participants

Seventy-one parents of mean age 41.1(+11.1) years participated in 7 focus group discussions (FGDs) (Supplementary Table). Among the participants, 55 (77%) were males and 16 (23%) were females. The mean age of the participants was 41.1+ 1.1 (range 26-69) years and 30 (42%) of them were in the age group of 30-39 years. In terms of employment, 19 (27%) were farmers, 14 (20%) were teachers, and 7 (12.7%) were HEWs. The majority of participants (53/71, 75%) had at least finished high school. The majority of participants (61/71, 86%) were Orthodox Christians (Table1).

***Focus Group Discussions***

Seven major themes emerged from the thematic analysis related to Knowledge: (1) Information on child eye health; (2) Knowledge about child eye problems; attitude related themes (3) Barriers to child eye care; Attitudes: (4) Use of eye glasses; and (5) Impact of childhood blindness; and Practice: (6) Treatment and prevention of CEH problems and (7) Cultural and traditional practices. Table 2 describes these themes, sub-themes and codes identified during analysis, which are further discussed below.

1.0 Information on child eye health

Most participants reported obtaining information on CEH from health professionals (during their visits to health facilities or during home-to-home visits) or the media, such as radio (mainly in the form of advertisements about eye health). They cited that they received instruction on: personal and environmental hygiene (particularly to washing hands frequently and keeping eyes clean); when to go to health facilities; avoiding bright light; and minimizing television time. However, almost all participants emphasized that they felt that information they had access to about CEH is inadequate.

**Table 1.** Characteristics of FGD participants from selected Kebeles in two woredas of Amhara Region

| **Variable** | | **n** | **%** | **mean (+ SD)** | **range** |
| --- | --- | --- | --- | --- | --- |
| **Total Study Participants** | | **71** | **100%** |  |  |
| **Sex** |  |  |  |  |  |
|  | Male | 55 | 77% |  |  |
|  | Female | 16 | 23% |  |  |
| **Age (years)** | |  |  | 41.1(+11.1) | 26-69 |
|  | 20-29 | 8 | 11% |  |  |
|  | 30-39 | 30 | 42% |  |  |
|  | 40-49 | 18 | 25% |  |  |
|  | 50-59 | 7 | 10% |  |  |
|  | 60-69 | 8 | 11% |  |  |
| **Kebele/Neighbourhood** | |  |  |  |  |
|  | Aba Jale Kebele 11 | 13 | 18% |  |  |
|  | Adebabay Iyesus 04 | 12 | 17% |  |  |
|  | Shwa Ber Kebele 18 | 12 | 17% |  |  |
|  | Azezo Kebele 20 | 9 | 13% |  |  |
|  | Arbaya | 7 | 10% |  |  |
|  | Worehala | 9 | 13% |  |  |
|  | Kalay | 9 | 13% |  |  |
| **Education** | |  |  |  |  |
|  | Illiterate | 5 | 7% |  |  |
|  | Read and write | 6 | 8% |  |  |
|  | Elementary school | 7 | 10% |  |  |
|  | High School | 26 | 37% |  |  |
|  | Diploma and above | 27 | 38% |  |  |
| **Religion** | |  |  |  |  |
|  | Orthodox Christian | 61 | 86% |  |  |
|  | Muslim | 10 | 14% |  |  |
| **Employment Status** | |  | 0% |  |  |
|  | Employed | 68 | 96% |  |  |
|  | Farmer | 19 | 27% |  |  |
|  | Teacher | 14 | 20% |  |  |
|  | Local Leader | 8 | 11% |  |  |
|  | Government Employee | 7 | 10% |  |  |
|  | Health Extension Workers | 7 | 10% |  |  |
|  | Priest | 7 | 10% |  |  |
|  | Sheikh | 5 | 7% |  |  |
|  | Leader of Youth Association | 1 | 1% |  |  |
|  | Unemployed | 3 | 4% |  |  |
|  | Housewife | 2 | 3% |  |  |

**Table 2. Themes, sub-themes and representative quotes**

|  | **Themes** | **Subthemes** | **Quotes** |
| --- | --- | --- | --- |
| **KNOWLEDGE** | Information on child eye health | Source of information | We get the information about eye health on television and radio |
|  |  |  | I am a religious leader. From what I observed, most of the time we get the information about the disease and eye care from a health center |
|  |  |  | those who was treated and get cured from the illness tell us about it |
|  |  |  | Information on child eye health is obtained from health professionals working in under 5 children and their treatment history |
|  |  | Type of information | They received instruction on: personal and environmental hygiene (particularly to washing hands frequently and keeping eyes clean); |
|  |  |  | Information on when to go to health facilities; avoiding bright light; and minimizing television time. |
|  |  |  | Information on avoiding bright light; and minimizing television time. |
|  | Knowledge about child eye problems | Cause of eye problems | when children see into the dark their eyes tend to deviate |
|  |  |  | We educate the society on vision decrement caused by trachoma |
|  |  |  | Reduction of vision could be due to trachoma, dirt, and a lot of other diseases. |
|  |  |  | Deviation of the eyes in young children mostly arises from dirt, if they don’t wash their faces two to three times per day |
|  |  |  | Visual decrement is usually caused by seeing water and glowing things |
|  |  | Features of reduction in vision in children | if they have decreased vision, it can be treated at hospital |
|  |  |  | When our visual ability decreases, we have difficulties to read distant writings. |
|  |  |  | Children fall when walk |
|  |  |  | Children in school can't properly copy |
|  | Barriers to child eye care | Challenge to get services | Poor attention is given to children eye care |
|  |  |  | The major thing is due to a lack of knowledge they don't think it can be treated. |
|  |  |  | One is financial constraint, because of it many with curable disease failed to get treatment. |
|  |  |  | some who seek for traditional treatment because they cannot afford to go to a health center |
|  |  |  | Financial constraint, and most of them live far and there is no access for transportation. |
|  |  | Health institution related factors | Parents lose hope when they become appointed after visiting medical centers. |
|  |  |  | Even at Gondar hospital there is very long appointment for services |
|  |  |  | the health centers is substandard it will be like the pig story |
|  |  |  | Within the nearby hospital (Arbaya) no specialist eye Doctor |
| **ATTITUDES** | Use of eye glasses | Attitude towards eyeglass use | our tradition and thinking is that wearing glasses for children brings harm to them. |
|  |  |  | When people see children wearing glasses they might say why is he wearing a glass he's just a kid |
|  |  |  | they may also not play as much as the other kids cause of the fear that the glasses will break. |
|  |  |  | Those children that wear glasses might be out casted |
|  |  |  | If children wear their glasses and go to school they will be insulted and so they don’t wear |
|  |  | Importance of wearing eye glasses | especially in students the impact it has is being able to differentiate letters from a far and near |
|  |  |  | the treatment should continue as it is and glasses are mandatory for children |
|  | Treatment and prevention of CEH problems | Importance of personal hygiene | To prevent reduction of vision the major thing is maintaining their hygiene |
|  |  | Eating heathy diet | Eating a balanced diet (e.g. eating carrots was mentioned specifically) for preventing visual impairment |
|  |  | Avoid reading in sunlight | Avoiding reading in sunlight can prevent visual problems in children. |
| **PRACTICE** | Impact of childhood blindness | Thinking about the future | children may fall when walking if they have decreased vision |
|  |  |  | economic difficulty is also another problem with blindness |
|  |  |  | Children can have be labeled as “stupid”. |
|  | Cultural & traditional practices | Cultural issues | Alemochie’ means a word to describe when children develop fever, body rash, and red eyes, they |
|  |  |  | Kindibat is usually done by cutting the eye lid and its treatment for red eye. |
|  |  |  | Apply eye liners to newborn is one way to prevent eye diseases in children. |
|  |  | Traditional Healers | Some people seek for traditional treatment because they cannot afford to go to health facilities |
|  |  |  | Some go towards traditional medicines. |

For some of the study participants, sick persons were considered a source of health information. One participant emphasized that the advantage this approach was that:

*“...getting an information from a patient is good because we can have complete information from that person, including prevention means and where we can get treatment if we need it.” –Local leader.*

2.0 Knowledge about child eye problems

*2.1. Cause of eye problems*

The participants outlined trachoma, glaucoma, exposure to dirt particles, and trauma to be the causes of poor eyesight in children. Misaligned eyes (strabismus) was another condition that participants mentioned as commonly observed in their communities. Regarding the cause of strabismus almost all of them mentioned “staring at light” as the cause. One participant said;

*“Traditionally our mothers would say when children see into the dark their eyes tend to deviate. This can be avoided by preventing the children’s view by blocking cloth or a curtain so they wouldn’t see towards bright lights at night.” -Priest.*

*2.2. Features of reduction in vision in children*

The participants believed that children with poor eyesight would have difficulty seeing at night and in reading. They described the need to sit at the front of the classroom or near the television to see more clearly. However, almost all participants didn’t know when they would seek care for such issues. One of the parent participants, who also worked as a HEW, said:

*“I personally have found many children who had squint, but I had no idea if it was treatable. And hence, I have not told them to seek treatment. But I know if they have decreased vision, it can be treated at hospital”.* -HEW

Regarding seeking child eye care, the majority of the study participants mentioned “pain and redness” as a reason for seeking treatment. Another participant described his take as:

*“Eye treatment needs high level of care, but we have little knowledge and we do not seek medical care early. When our visual ability decreases, we have difficulties to read distant writings. We need to take a closer look to read. Especially when we have tear or burning sensations to our eyes, we need to seek eye treatment”. -Teacher.*

3.0 Barriers to child eye care

Most participants put financial issues (inability to afford treatment) and lack of awareness as forerunners that affect access to child eye care. Several participants also raised health provider-related issues, including absence of the correct health professional (e.g. eye specialist), very long wait times, and referral to facilities far from their home (usually to the University of Gondar) as barriers to accessing CEH services.

Some also raised the issues of lack of transportation, less emphasis by government on delivery of CEH services, and limited media attention to CEH issues, as barriers to access CEH service. One participant stated his dismay as follows:

*“Poor attention is given to children eye care. Starting from clinics and hospitals including health minister give poor attention to children eye care and I believe this is one obstacle. In seeing the society there could also be limitation in the awareness”.*  - Leader of Youth Association.

Another participant, described his concern about health provider quality as follows:

*“I am sure you know what a pig is. A pig comes out of the woods intentionally to wash itself, but after it’s done washing it rolls in the mud. If the medias are supported financially and they present the eye health need well, but if the service at the health centers is substandard it will be like the pig story”. -Teacher*

4.0 Use of eye glasses

Some participants perceived wearing spectacles as useful, as they improve vision (e.g., important for reading), protect eyes from dirt and bright lights and protect from illness. However, the majority of the participants mentioned stigma against children wearing spectacles as a concern.

One participant said:

*“I believe wearing glasses has benefits and no harm. In kids usually wearing glasses is seen as being lame and being outcasted by the other students and this is due to lack of awareness”. -Teacher.*

Some of the participants had concerns about the use of eyeglasses for children, as they had limited experience with children wearing spectacles. One participant discussed his concern as follows:

“*Till now I* *have never seen or heard anybody teaching children to use eyeglasses. And I believe it will be better if the health care givers get a lot involved in creating awareness”. -Teacher.*

Some of them even believe that vision of children wearing spectacle will deteriorate. And another participant said:

*“People advise against routine use of eyeglasses because they believe it decreases vision.* *If the eyeglass is given by a professional after thorough examination, they will use it. The main thing is, I believe that attention should be given to other treatments and glasses be made the last resort and that other treatments are given to maintain their eyesight, keep their hygiene, but the main thing is that the health professional creates awareness at the lower levels including woreda and kebeles.” -Sheikh*

Another participant put his opinion towards children wearing spectacles as:

*“My personal attitude towards using eyeglasses in children is, if someone in my family is prescribed eyeglasses, I am not willing because it will be a bad thing if they start to wear glasses from this age. If they start to wear glasses at a young age, they could be short sighted”. -Farmer.*

**5.0 Impact of childhood blindness**

The impact of visual impairment on children was outlined by the participants as poor school performance and repeatedly failing to progress. They also said children would sustain psychological trauma as the result of insults from their peers and teachers and being labeled as “stupid”.

One of the school teacher described the following regarding the impact of poor vsion in education as:

*“Visual problems by themselves are one of the reasons for poor school performance. One of my students had terrible handwriting and struggled to keep up with the blackboard. Her results were dismal, and she failed that academic year. She keeps asking to be in the front chair, she mentioned she can't see from a distance. When eye doctors came to our school for a free eye exam, I took her for an eye exam. They also informed us that she requires glasses. Sadly, her poor performance was due to her eye problem.”. –Teacher*

6.0 Treatment and prevention of child eye health problems

The participants stated the importance of keeping good personal and environmental hygiene and eating a balanced diet (e.g. eating carrots was mentioned specifically) for preventing visual impairment in children. They also indicated that eye health education and messaging was important for promoting the use of these practices.

When it came to seeking care, none of the participants had taken their children to receive an eye examination. Most participants said they would seek treatment specifically if their children’s vision was reduced. One participant said:

*“To prevent reduction of vision the major thing is maintaining their hygiene. The other thing is going to health centers. We can’t say that eye care is fully available in the society. For example, eye care is given at a hospital level in only one institution and so that the children of the community have access to this, there should be some reforms.”-Teacher*

And another participant expressed his position as follows:

*“I tell my children not to read in sunlight and try to keep their personal hygiene Promoting the habit of using eyeglasses, avoiding reading in sunlight and keeping hygiene can prevent eye diseases.” -Government employee.*

**7.0 Cultural & traditional practices**

Participants spoke about childhood diseases that have specific cultural names, perceived causes and traditional treatment. “Alemochie” is a cultural nomenclature for measles and traditionally it has its own treatment, described as follows by one participant:

*“*Alemochie*’ means a word to describe when children develop fever, body rash, and red eyes, they mix incense, niger, and roasted barley, after which they will climb a tree and a traditional custom starts.” –woman Local leader.*

For some of the study participants, traditional medicine was easily accessible and affordable. One participant described his observation on the cost of eye care and waiting time at health facilities as follows:

*“Some people seek for traditional treatment because they cannot afford to go to health facilities. And even if they go, they spend hours in the waiting room waiting for their turn before they get the treatment. So, I think it will be better if there is a separate institution* *for treating these conditions.” -HEW*

Primary health care workers participated in this study mentioned that vising traditional healers is a common practice among parents. One of the HEW said:

*“We tell the mothers to take the children to the hospital to be evaluated, but some go towards traditional medicines.” -HEW*

DISCUSSION

**This qualitative study in the Northwest part of Ethiopia revealed gaps in parental knowledge and attitude towards child eye health, as well as practices that may interfere with prevention and treatment of visual impairment and blindness in children. Such qualitative studies have the potential to explain the nature and kind of barriers in child eye health [24].**

An estimated 100,000 blind youngsters reside in Ethiopia alone. According to a 2017 study at a blind school in the same region as the current study, corneal scarring from vitamin A deficiency and measles are the leading causes of blindness [25]. Yet these were not noted by the participants in our study, who instead identified trachoma, glaucoma, and trauma as the primary causes of childhood blindness. Furthermore, none of the participants mentioned amblyopia as a childhood vision concern. This finding is consistent with research from India [26], and Nigeria [28] where in each study, only one parent was aware of amblyopia. In contrast, a research study from Saudi Arabia revealed that parents' understanding of amblyopia was the greatest among other disorders [29]. Amblyopia can be prevented and treated, particularly if it is detected early in children under the age of eight [30].

The primary sources of information, according to study participants, were the media and health professionals. However, they all acknowledged that they lack sufficient knowledge regarding children's eye health. This may be the cause of parents misunderstanding the etiologies and common causes of child eye blindness in their setting. People in Eswatini (formerly Swaziland), rely on doctors and nurses for knowledge about eye health [31]. In order to overcome the conventional hurdles to access health care, such as geography, finance, and cultural considerations, some have argued in favor of using electronic devices to distribute health information [32]. The increase in the internet penetration rate from 7.5 to 25% in the past 10 years in Ethiopia gives an opportunity to use the internet as a source of health information in the coming years [33]. However, such activities should be coupled with campaigns to educate the public on trustworthy sources of information, such as the ministry of health, such that misinformation is avoided. Also, people with lived experience of vision impairment or blindness could be another knowledgeable and trusted source of information, as noted by one participant in our study. Health champions have been instrumental in raising awareness for HIV/AIDS, and could have an equivalent role here.

This study revealed a myth in the community related to strabismus causation, where parents believed that strabismus was caused by staring at light. A prior cross-sectional community survey in Southern Ethiopia revealed that many of the participants had false beliefs regarding what causes strabismus [36]. Similarly, a study carried out in Nigeria demonstrated that a common misconception was that strabismus could result "if a baby's sleep is not changed from time to time" [37]. A study from Chennai, India indicated that the majority of parents in that setting believed that strabismus was a sign of good fortune and did not seek care for it. [26]. However , strabismus can be the first sign of ocular tumor, like retinoblastoma [38]. Poor parental education, misunderstandings, and misinformation have a negative impact on the age of strabismus presentation and management, which differs from nation to country [39]. Misconceptions like these have been reported about other eye conditions; for example, a study in India revealed that parents considered that unhealthy eating habits and lack of proper nutrition caused ocular disorders like refractive error [26]. An earlier study conducted in Ethiopia revealed that there was a widespread perception in the community that childhood blindness may be due to curses [27].

This study revealed differences in perceptions among the study participants with regard to the use of spectacles in children. While some parents felt that wearing eyeglasses on a regular basis could impair vision, others believed that it had benefits including protection from dust and strong light and improving vision. However, the stigma associated with wearing glasses was a major worry raised by the majority of participants. Similarly, study from India showed that parents were hesitant to provide spectacle correction for their children with vision problems because they perceived wearing spectacles as a stigma [26]. According to Nirmalayan et al, participants in their study mentioned that no child under the age of four should be permitted to wear glasses at any time [40]. A study in Nigeria showed that many parents have misconceptions about their child's need for spectacles, believing that the child may become overly reliant on them and be unable to function without them [28]. Parental disapproval has been reported as one of the main reasons for the non-compliance with spectacle utilization among school students [41]. Identifying reasons for non-compliance with spectacle use is important for understanding the social determinants of intervention. This will help to enhance the awareness of the parents of children with refractive error, by informing health education at the respective health facilities.

Despite the above misconceptions and gaps in knowledge, participants in the study were able to recognize signs of children's poor vision. They also were aware of negative effects of poor vision, noting how children with visual impairment tend to do poorly in school and repeatedly fail to progress. Similar findings have been shown in studies from Eswatini, Nigeria, Saudi Arabia and India [26, 28, 29]. Yet, our participants were unable to pinpoint the right moment to seek medical assistance. None of the participants mentioned taking their children to the eye doctor for routine checkups. A European study of parental health-seeking behavior indicates that upon first notice of signs and symptoms parents initially attempt to manage the condition at home, and tend to seek medical attention when they feel they have “lost control of the situation” [50]. Poor parental health-seeking behavior has been attributed to a lack of health information in prior research by Muhit et al. [12] and Shrestha et al. [34]. In Ethiopia, there is currently no specific health protocol / infrastructure for detection and management of childhood blindness in primary and secondary health care systems [22]. Thus, it is the parents' responsibility to take care of their children's eyes. As the primary caregivers, parents decide when and if to seek out medical care for their children [35].

When parents do decide to seek care, they face several barriers in the Ethiopian context. Financial constraints, a lack of public knowledge, a shortage of qualified eye health specialists, lengthy wait times at tertiary eye care facilities, and a disregard for children's eye health were highlighted as barriers to obtaining child eye health in our study. This is consistent with the literature showing that non-availability, non-accessibility, and non-affordability of eye care are three primary factors that contribute to the high prevalence of childhood visual impairment in Africa [2, 13, 14, 16. 28, 31, 42 -48].

Our study highlighted that parents' health-seeking behaviors’ were affected by substandard health facilities. Numerous researches have revealed that socioeconomic position, access to healthcare facilities, and perceived service quality are important determinants of health seeking behavior among different population groups [49-51]. Therefore, improving the health seeking behavior of parents must go hand in hand with improving capacity to provide excellent child eye care.

Furthermore, ineffective policies and a lack of facilities have also been mentioned as a major barrier for child eye health, particularly in low-income nations [2]. Perhaps then, as a response to these barriers, traditional medicine was identified as one of the treatment alternatives by the study's participants. Similarly, a study from Nigeria revealed that parents frequently used local or traditional medicine [28]. Aside from the general public's access and affordability to eye health, deep-rooted cultural beliefs and misconceptions about the causes of childhood illnesses may be reasons for a preference for traditional healers. Parental knowledge tends to dominate decisions when there is discordance between professional and lay knowledge [52].

**CONCLUSION**

In conclusion, this study captured the current state of knowledge, attitude and practice towards child eye health among parents in Northwest Ethiopia. The study revealed concerning gaps in knowledge and incorrect views on strabismus, spectacle utilization and the etiologic causes of the most common childhood eye diseases. Study participants revealed a desire for more health information on child eye diseases from trusted sources, like health professionals and patients.

As the majority of childhood blindness causes are either avoidable or preventable, the study suggests that raising parental awareness of the most common child eye conditions may improve early detection and facilitate appropriate management. Increasing community awareness, through social media campaigns endorsed by the Ministry of Health, may aid in reducing childhood blindness in Ethiopia.

***Acknowledgements***

We would like to thank Light for the World for providing financial support for this study. Our special appreciation goes to Suadik Hassen , Alem Mekonen, Eyoel Lemma Semalign Abew and Demeke Debebe for their wonderful support and facilitation. Salient gratefulness is extended to all the study participants, community representatives and others who participated in the discussions by providing all the necessary information for the study.

**Conflict of interest-** None

**Funding** – Light for the World financially supported the field work of this study.

***Authors’ Contributions***

Drafting of the manuscript: S.T.S., S.T,. Z.E., A.A., and H.D. ; revision of the manuscript for important intellectual content: S.T.S., S.T,. Z.E., A.A., and H.D..; conception and design of study: S.T.S., S.T,. Z.E., A.A., and H.D.; data acquisition, analysis or interpretation of data: S.T.S., S.T,. Z.E., A.A., and H.D..; approval of final manuscript to be published: S.T.S., S.T. , Z.E., A.A., and H.D. All authors have read and approved the final manuscript.

**REFERENCES**

Gilbert C, Foster A. Childhood blindness in the context of Vision2020—the right to sight. Bull World Health Organ 2001; 79:227–32.

Borrel A, Dabideen R, Mekonen Y, Overland L. The African Child Policy Forum. Cape Town: ORBIS Africa; 2013. Child eye health in Africa: The status and way forward; pp.

1-37.

Bronard, A., Geneau, R., Shirima, S., Courtright, P. and Mwendi, J. (2008). Why are children brought late for cataract surgery: Qualitative findings from Tanzania. Ophthalmic Epidemiology, 15(6): 383-388.

Lewallen, S. and Courtright, P. (2001). Blindness in Africa: present situation and future needs. British Journal of Ophthalmology, 85: 897-903.

Frick KD, Foster A. The magnitude and cost of global blindness: an increasing problem that can be alleviated. Am J Ophthalmol 2003; 135:471-6.

Wilson ME, Pandey SK, Thakur J. Pediatric cataract blindness in the developing world: Surgical techniques and intraocular lenses in the new millennium. Br J Ophthalmol 2003; 87:14-9.

Chandna, A. and Gilbert, C. (2010). When your eye patient is a child. Community Eye Health Journal, 23(72) March 2010.

Pascolini D, Mariotti SP. Global estimates of visual impairment: 2010. Br J Ophthalmol 2012;96:6148.

World Health Organization, Geneva, 1999. Preventing blindness in children. WHO/PBL/00.77.

Dandona R, Dandona L, John RK, McCarty CA, Rao GN. Awareness of eye diseases in an urban population in southern India. Bull World Health Org. 2001; 79(2):96-102.

World Health Organization (WHO). Vision 2020. The Right to Sight. VISION 2020 Action Plan 2006-2010.

Muhit MA, Shahjahan M, Hassan A, Wazed A, Ahmed N. Parental knowledge, attitude and practice related to blindness of children in some selected Upazilla of Bangladesh. Mymensingh Med J. 2011; 20(4):671-679.

Alrasheed SH. A systemic review of barriers to accessing paediatric eye care services in African countries. African Health Sciences. 2021 Dec 14;21(4):1887-97.

Ntsoane MD, Oduntan OA, Mpolokeng BL. Utilisation of public eye care services by the rural community residents in the Capricorn district, Limpopo Province, South Africa. African journal of primary health care & family medicine. 2012;4(1).

Ramai D, Pulisetty T. Maternal and Caregiver Perceptions to Childhood Eye Care In Ghana. Internet J Epidem. 2013; 11(1): 2- 8.

Balasubramaniam SM, Kumar DS, Kumaran SE, Ramani KK. Factors affecting eye care – seeking behavior of parents for their children. Optom Vis Sci. 2013; 90 (10): 1138-42.

Kovai V, Krishnaiah S, Shamanna BR, Thomas R, Rao GN. Barriers to accessing eye care services among visually impaired populations in rural Andhra Pradesh, South India. Indian J. Ophthalmol. 2007; 55: 365-371.

AmiebenomoOM, Amiebenomo HO, Emadamerho OL. A hospital based study of traditional eye medicines usage in Benin City, Edo State. J Nig Optom Assoc.2014; 19(1): 12-16.

Van Dijk K, Courtright P. Barriers to surgical intervention among blind and low vision children in Malawi. Visual Impairment Research. 2000 Jan 1;2(2):75-9.

Health & Health-Related Indicators, 2011 EFY (2018/19.

Berhane Y, Worku A, Bejiga A,Adamu L, Alemayehu W, Bedri A, Haele, Z, Ayalew A, Amadu Y, Gelore T, Kebede DWest S. . Prevalence and causes of blindness and low vision in Ethiopia, Journal of Health Development.2007; 21(3): 204-210.

https://www.statsethiopia.gov.et/population-projection/.

Charmaz K. Constructing grounded theory - 2nd edition. London, UK: Sage Publications Ltd. 2014.

Bowman RJ. How should blindness in children be managed? Eye. 2005 Oct;19(10):1037-43.

Asferaw M, Woodruff G, Gilbert C. Causes of severe visual impairment and blindness in students in schools for the blind in Northwest Ethiopia. BMJ global health. 2017 Jun 1;2(2):e000264.

Senthilkumar DS, Balasubranmaniam MS, Kumaran EK, RamaniK. Parents’ awareness and perception of children’s eye diseasesin Chennai. India Optom Vis Sci. 2013;90:1462-1466

Belaynew WT, Berihun MZ, Tadesse AA, Yared AW. Knowledge and practice on childhood blindness among communities in Northwest Ethiopia: implications to blindness prevention programs. Journal of Ophthalmology. 2013.

Ebeigbe JA, Emedike CM. Parents’ awareness and perception of children's eye diseases in Nigeria. Journal of Optometry. 2017 Apr 1;10(2):104-10.

Surrati AM, Almuwarraee SM, Mohammad RA, Almatrafi SA, Murshid SA, Khayat LI, Al-Habboubi HF. Parents’ Awareness and Perception of Children’s Eye Diseases in Madinah, Saudi Arabia: A Cross-Sectional Study. Cureus. 2022 Feb 25;14(2).

Webber AL. Amblyopia treatment: an evidence‐based approach to maximizing treatment outcome. Clinical and Experimental Optometry. 2007 Jul 1;90(4):250-7.

Sukati VN, Moodley VR, Mashige KP. Knowledge and practices of parents about child eye health care in the public sector in Swaziland. African Journal of Primary Health Care and Family Medicine. 2018 May 3;10(1):1-3.

Gresenz C, Rogowski JA, Escarce J. Social networks and access to health care among Mexican-Americans.

Internet World Statistics: Usage and Population Statistics,Available at: http://www.internetworldstats.com/stats1.htm).

Shrestha MK, Guo CW, Maharjan N, Gurung R, Ruit S. Health literacy of common ocular diseases in Nepal. BMC ophthalmology. 2014 Dec;14(1):1-8.

Glascoe FP. Parents' evaluation of developmental status: how well do parents' concerns identify children with behavioral and emotional problems?. Clinical pediatrics. 2003 Mar;42(2):133-8.

Geta K, Bejiga A. Knowledge, attitude and practice towards strabismus in Cheha District, Central Ethiopia. Ethiopian Journal of Health Development. 2011;25(3):212-5.

Bodunde O, Runsewe-Abiodun T, Alabi A, Adetunji J, Olowonyo M, Adefuye B. . Awareness and Perception of Strabismus Among the Youths and Women of Child-Bearing Age in a Local Government in South Western Nigeria: A Qualitative Study. International Quarterly of Community Health Education, 2016; 37(1), 21–25. https://doi.org/10.1177/0272684X16685248.

Kiernan M, Fabian ID, Smith V, Sagoo MS, Reddy MA. Strabismus as a Presenting Sign in Retinoblastoma. Journal of Pediatric Ophthalmology & Strabismus. 2021 Sep 1;58(5):324-30.

Eustis S, Smith DR. Parental understanding of strabismus. Journal of Pediatric Ophthalmology & Strabismus. 1987 Sep 1;24(5):232-6.

Nirmalayan P, Sheeladevi D, Tamilselvi S, Victor A, Vijayalakshmi P, Rahmathulla L. Perception of eye disease and eye care needs of children among parents in rural South India: The Kariapatti pediatric eye evaluation project (KEEP). Comm Eye Care. 2004;2:1.

Dhirar N, Dudeja S, Duggal M, Gupta PC, Jaiswal N, Singh M, Ram J. Compliance to spectacle use in children with refractive errors-a systematic review and meta-analysis. BMC ophthalmology. 2020 Dec;20(1):1-1.

Adio AO, Komolafe RD. The State of Paediatric Eye Care in Nigeria: A Situational Review and Call for Action. Nigerian Health Journal. 2013;13(1):1-6.

Gogate P, Kalua K, Courtright P. Blindness in childhood in developing countries: time for a reassessment. PLoS Medicine. 2009 Dec 8;6(12): e1000177.

Agarwal PK, Bowman R, Courtright P. Child eye health tertiary facilities in Africa. Journal of American Association for Paediatric Ophthalmology and Strabismus. 2010 Jun 1;14(3):263-266.

Silva JC, Bateman JB, Contreras F. Eye disease and care in Latin America and the Caribbean. Survey of Ophthalmology. 2002 May 1;47(3):267-274.

Fafowora OF. Prevalence of blindness in a rural ophthalmically underserved Nigerian community. West African Journal of Medicine. 1995 Dec;15(4):228-231.

Robin AL, Nirmalan PK, Krishnadas R, Ramakrishnan R, Katz J, Tielsch J, Thulasiraj RD, Friedman DS. The utilization of eye care services by persons with glaucoma in rural south India. Transactions of the American Ophthalmological Society. 2004 Dec; (102):47-55.

Naidoo K. Poverty and blindness in Africa. Clinical and Experimental Optometry. 2007 Nov 1;90(6):415-421.

Phiri SNa, Kiserud T, Kvale G, et al. Factors associated with health facility childbirth in districts of Kenya, Tanzania and Zambia: a population based survey. BMC pregnancy and childbirth. 2014;14(1):219.

Babalola S, Fatusi A. Determinants of use of maternal health services in Nigeria-looking beyond individual and household factors. BMC pregnancy and childbirth. 2009;9(1):43.

Gao W, Dang S, Yan H, Wang D. Care-seeking pattern for diarrhea among children under 36 months old in rural western China. PloS one. 2012;7(8):e43103.

Britten N, Stevenson FA, Barry CA, Barber N, Bradley CP. Misunderstandings in prescribing decisions in general practice: qualitative study. Bmj. 2000 Feb 19;320(7233):484-8.

***Figures Legend***

Table 1. Characteristics of FGD participants from selected Kebeles in two woredas of Amhara Region

Socio-demographic characteristics of the focus group discussion participants.

Table 2. *Themes, sub-themes and representative quote*
