# Supplementary material for: Exploring the knowledge, attitudes, and practice towards child eye health: A qualitative analysis of parent experience focus groups
Source: PLoS One. 2023 Nov 3;18(11):e0293595. doi: 10.1371/journal.pone.0293595 (PMC10624311; doi:10.1371/journal.pone.0293595)
Supplement: Attachment — Submitted filename: Comments to Author.docx [file pone.0293595.s004.docx]

The purpose of the study has been clearly stated to report on the knowledge, attitudes and practice among parents towards CEH in Ethiopia.

There is need to further expatiate on the justification for this study with particular emphasis on why a qualitative approach and what this would buttress to the study.

The study also needs to highlight past studies on knowledge, attitudes and practice among parents towards CEH in Ethiopia and state what differentiates this study from others or state if there is paucity of such studies.

The methodology has been well explained, however a few concerns.

There are concerns to the fact that a purposive sampling was used to select the participants but there clearly seems to be a selection bias towards males as there were 55 males and 16 females. In the African context , females are usually seen to be the ones that take care of the home and the children at home , so its preferable that there should be a fair representation of both sexes .

Also because of the family dynamics and cultural practice in Arica, its advisable that females and males have separate focus group discussion, so females can be able to express themselves better as a number of African cultures , women have been seen not to fully express their views or be open to speak freely in the presence of males.

Details of the methodology are sufficient and some recommendations above should be considered especially towards subsequent studies.

The manuscript was well written and clear enough to be accessible to non-specialist .

The is need for the authors to conform to a uniform referencing style as recommended by the journal and a few references were found not to be complete.
